# Supplementary material for: Principal component conditional generative adversarial networks for imbalanced ECG classification enhancement
Source: PLoS One. 2025 Aug 22;20(8):e0330707. doi: 10.1371/journal.pone.0330707 (PMC12373255; doi:10.1371/journal.pone.0330707)
Supplement: Attachment — Submitted filename: COMMENTS.pdf [file pone.0330707.s002.pdf]

COMMENTS

In this paper, the authors discusses the core challenges in ECG signal classification—extremely imbalanced data, significant individual physiological differences, and difficulties in long sequence fitting—by proposing a Principal Component Analysis-based Conditional Generative Adversarial Network (PCA-CGAN).

The author has claimed as innovations:
(i)Paradigm Shift in ECG Signal Representation:
(ii)Transformer-based Conditional Generative Adversarial Network
(iii)Two-Stage Conditional Encoding-Decoding Strategy
(iv)Resolving the Dilution Effect in Data Augmentation:
(v)Breaking the Single-Patient Limitation to Achieve Multi-Patient ECG Signal Generation:

The comparison of the claimed approaches with existing literature, as well as performance evaluation, are not sufficiently detailed. There is a lack of quantitative performance comparison and analysis of pros and cons in practical applications.

"The coining of sub titles for each session should be in a more precise manner" suggests that the current subtitles are not clear or descriptive enough. To improve them, consider using more specific and informative language that accurately reflects the content of each session.

There are numerous instances of English grammatical errors and awkward expressions.
