# Supplementary material for: Principal component conditional generative adversarial networks for imbalanced ECG classification enhancement
Source: PLoS One. 2025 Aug 22;20(8):e0330707. doi: 10.1371/journal.pone.0330707 (PMC12373255; doi:10.1371/journal.pone.0330707)
Supplement: Attachment — Submitted filename: Response to Reviewers.docx [file pone.0330707.s004.docx]

**Reviewer #1:** In this paper, the authors discusses the core challenges in ECG signal classification—extremely imbalanced data, significant individual physiological differences, and difficulties in long sequence fitting—by proposing a Principal Component Analysis-based Conditional Generative Adversarial Network (PCA-CGAN).

The author has claimed as innovations:
(i)Paradigm Shift in ECG Signal Representation:
(ii)Transformer-based Conditional Generative Adversarial Network
(iii)Two-Stage Conditional Encoding-Decoding Strategy
(iv)Resolving the Dilution Effect in Data Augmentation:
(v)Breaking the Single-Patient Limitation to Achieve Multi-Patient ECG Signal Generation:

1.The comparison of the claimed approaches with existing literature, as well as performance evaluation, are not sufficiently detailed. There is a lack of quantitative performance comparison and analysis of pros and cons in practical applications.

2."The coining of sub titles for each session should be in a more precise manner" suggests that the current subtitles are not clear or descriptive enough. To improve them, consider using more specific and informative language that accurately reflects the content of each session.

3.There are numerous instances of English grammatical errors.

**Response to Reviewer #1**

**1. Comparison with existing literature and performance evaluation**

**Thank you for pointing out this critical weakness in our manuscript. We fully agree that our initial submission lacked sufficient comparative analysis. Following your valuable guidance, we have substantially expanded our literature review and added comprehensive experimental analyses comparing our approach with existing methods. We have incorporated extensive experimental analyses comparing our PCA-based approach with other dimensionality reduction techniques, and added detailed quantitative analysis throughout the manuscript.**

**2. Improvement of section titles**

**We sincerely apologize for the unclear organization and section titles in our original submission. Your comment helped us realize the importance of clear structural presentation. We have restructured the manuscript using a hierarchical heading system that clearly distinguishes between major sections and subsections, making the overall structure much clearer for readers.**

**3. English grammatical errors**

**We deeply apologize for the numerous grammatical errors in our initial submission. This was indeed unprofessional, and we take full responsibility. We have conducted a thorough proofreading of the entire manuscript and corrected all identified grammatical errors and potential issues. We hope the revised version meets the journal's standards.**

**Reviewer #2:** Areas for Improvement / Suggestions
1.Clarity and Language Polishing
While the technical content is strong, the manuscript would benefit from language editing for clarity and conciseness. Some expressions are repetitive, and certain technical terms (e.g., “Matthew effect,” “dilution effect”) could be introduced more formally with brief definitions for clarity.

2.Ablation Studies Needed
To better isolate the impact of each innovation (PCA, conditional encoding, Transformer), include ablation studies showing performance degradation when specific components are removed or replaced.

3.Broader Comparison with Existing Methods
While SigCWGAN and TD-GAN are compared, additional benchmarking against more recent diffusion models or contrastive learning approaches would further contextualize your model’s effectiveness.

4.Discussion on Limitations and Clinical Translation
The paper should include a brief section acknowledging limitations (e.g., reliance on synthetic data, assumptions in PCA), and a forward-looking discussion on how this work can transition into clinical ECG diagnostic tools.

5.Over-Reliance on PCA Justification
Although the use of PCA is well-motivated, future directions could explore non-linear dimensionality reduction techniques (e.g., autoencoders or t-SNE embeddings) to validate or enhance the representational efficiency.

6.Ethical and Reproducibility Considerations
A brief statement on ethical use, model reproducibility, and potential biases (e.g., how patient diversity was handled) would be welcome in keeping with best practices for clinical AI research.

**Response to Reviewer #2**

**1. Clarity and technical term definitions**

**You are absolutely right that technical terms should be properly introduced. We apologize for this oversight. Technical terms such as "Matthew effect" and "dilution effect" are now formally introduced with clear definitions and explanations before their discussion in the text.**

**2. Ablation studies**

**Thank you for this excellent suggestion. We realize that ablation studies are crucial for validating our contributions. Following your guidance, we have conducted comprehensive ablation studies, analyzing the performance changes when each component is removed.**

**3. Broader comparison with existing methods**

**Your point about needing broader comparisons is well-taken. We have added more in-depth descriptions and comparisons of various model types, and included comprehensive discussions on different approaches.**

**4. Discussion on limitations**

**We greatly appreciate you highlighting the need for a balanced discussion of limitations. At the end of the paper, we have added a section candidly describing the model's limitations, discussing both its advantages and disadvantages.**

**5. Non-linear dimensionality reduction techniques**

**Thank you for suggesting this important comparison. We have added discussions and comparisons of other non-linear dimensionality reduction techniques, analyzing the advantages, disadvantages, and applicable scope of each technique.**

**6. Code and data availability**

**Following your recommendation for reproducibility, we have included the paper's code and datasets in the submission attachments to facilitate better understanding and reproduction of our work.**
