# Supplementary material for: Principal component conditional generative adversarial networks for imbalanced ECG classification enhancement
Source: PLoS One. 2025 Aug 22;20(8):e0330707. doi: 10.1371/journal.pone.0330707 (PMC12373255; doi:10.1371/journal.pone.0330707)
Supplement: Attachment — Submitted filename: Response to Reviewer 2.docx [file pone.0330707.s005.docx]

**Reviewer #2:** Areas for Improvement / Suggestions
1.Clarity and Language Polishing
While the technical content is strong, the manuscript would benefit from language editing for clarity and conciseness. Some expressions are repetitive, and certain technical terms (e.g., “Matthew effect,” “dilution effect”) could be introduced more formally with brief definitions for clarity.

2.Ablation Studies Needed
To better isolate the impact of each innovation (PCA, conditional encoding, Transformer), include ablation studies showing performance degradation when specific components are removed or replaced.

3.Broader Comparison with Existing Methods
While SigCWGAN and TD-GAN are compared, additional benchmarking against more recent diffusion models or contrastive learning approaches would further contextualize your model’s effectiveness.

4.Discussion on Limitations and Clinical Translation
The paper should include a brief section acknowledging limitations (e.g., reliance on synthetic data, assumptions in PCA), and a forward-looking discussion on how this work can transition into clinical ECG diagnostic tools.

5.Over-Reliance on PCA Justification
Although the use of PCA is well-motivated, future directions could explore non-linear dimensionality reduction techniques (e.g., autoencoders or t-SNE embeddings) to validate or enhance the representational efficiency.

6.Ethical and Reproducibility Considerations
A brief statement on ethical use, model reproducibility, and potential biases (e.g., how patient diversity was handled) would be welcome in keeping with best practices for clinical AI research.

**Response to Reviewer #2**

**1. Clarity and technical term definitions**

**You are absolutely right that technical terms should be properly introduced. We apologize for this oversight. Technical terms such as "Matthew effect" and "dilution effect" are now formally introduced with clear definitions and explanations before their discussion in the text.**

**2. Ablation studies**

**Thank you for this excellent suggestion. We realize that ablation studies are crucial for validating our contributions. Following your guidance, we have conducted comprehensive ablation studies, analyzing the performance changes when each component is removed.**

**3. Broader comparison with existing methods**

**Your point about needing broader comparisons is well-taken. We have added more in-depth descriptions and comparisons of various model types, and included comprehensive discussions on different approaches.**

**4. Discussion on limitations**

**We greatly appreciate you highlighting the need for a balanced discussion of limitations. At the end of the paper, we have added a section candidly describing the model's limitations, discussing both its advantages and disadvantages.**

**5. Non-linear dimensionality reduction techniques**

**Thank you for suggesting this important comparison. We have added discussions and comparisons of other non-linear dimensionality reduction techniques, analyzing the advantages, disadvantages, and applicable scope of each technique.**

**6. Code and data availability**

**Following your recommendation for reproducibility, we have included the paper's code and datasets in the submission attachments to facilitate better understanding and reproduction of our work. The complete implementation of PCA-CGAN, including training scripts, evaluation metrics, and preprocessing pipelines, is publicly available at:**

**https://github.com/ChaoTang20250509/PCA_CGAN**
