# Supplementary material for: Principal component conditional generative adversarial networks for imbalanced ECG classification enhancement
Source: PLoS One. 2025 Aug 22;20(8):e0330707. doi: 10.1371/journal.pone.0330707 (PMC12373255; doi:10.1371/journal.pone.0330707)
Supplement: Attachment — Submitted filename: Response to Reviewer 1.docx [file pone.0330707.s006.docx]

**Reviewer #1:** In this paper, the authors discusses the core challenges in ECG signal classification—extremely imbalanced data, significant individual physiological differences, and difficulties in long sequence fitting—by proposing a Principal Component Analysis-based Conditional Generative Adversarial Network (PCA-CGAN).

The author has claimed as innovations:
(i)Paradigm Shift in ECG Signal Representation:
(ii)Transformer-based Conditional Generative Adversarial Network
(iii)Two-Stage Conditional Encoding-Decoding Strategy
(iv)Resolving the Dilution Effect in Data Augmentation:
(v)Breaking the Single-Patient Limitation to Achieve Multi-Patient ECG Signal Generation:

1.The comparison of the claimed approaches with existing literature, as well as performance evaluation, are not sufficiently detailed. There is a lack of quantitative performance comparison and analysis of pros and cons in practical applications.

2."The coining of sub titles for each session should be in a more precise manner" suggests that the current subtitles are not clear or descriptive enough. To improve them, consider using more specific and informative language that accurately reflects the content of each session.

3.There are numerous instances of English grammatical errors.

**Response to Reviewer #1**

**1. Comparison with existing literature and performance evaluation**

**Thank you for pointing out this critical weakness in our manuscript. We fully agree that our initial submission lacked sufficient comparative analysis. Following your valuable guidance, we have substantially expanded our literature review and added comprehensive experimental analyses comparing our approach with existing methods. We have incorporated extensive experimental analyses comparing our PCA-based approach with other dimensionality reduction techniques, and added detailed quantitative analysis throughout the manuscript.**

**2. Improvement of section titles**

**We sincerely apologize for the unclear organization and section titles in our original submission. Your comment helped us realize the importance of clear structural presentation. We have restructured the manuscript using a hierarchical heading system that clearly distinguishes between major sections and subsections, making the overall structure much clearer for readers.**

**3. English grammatical errors**

**We deeply apologize for the numerous grammatical errors in our initial submission. This was indeed unprofessional, and we take full responsibility. We have conducted a thorough proofreading of the entire manuscript and corrected all identified grammatical errors and potential issues. We hope the revised version meets the journal's standards.**
