# Supplementary material for: Principal component conditional generative adversarial networks for imbalanced ECG classification enhancement
Source: PLoS One. 2025 Aug 22;20(8):e0330707. doi: 10.1371/journal.pone.0330707 (PMC12373255; doi:10.1371/journal.pone.0330707)
Supplement: Attachment — Submitted filename: Response_to_Reviewer_1_auresp_4.docx [file pone.0330707.s007.docx]

**Reviewer #1: The second suggestion given in my earlier comment "The coining of sub titles for each session should be in a more precise manner" suggests that the current subtitles are not clear or descriptive enough. To improve them, consider using more specific and informative language that accurately reflects the content of each session." has not been addressed properly.**

**The manuscript should have Introduction session.**

**Rename " Related Work and Problem Analysis" as "Introduction"**

**Rename "Conclusions and Technical Limitations" as "Discussions" and include relevant details into it. Also add more contents to discussion section incorporating your inference from comparisons and analysis.**

**Conclusion session should be brief and should contain only two paragraphs including the future work.**

**A separate session on "Related Works" should be added just after "Introduction" session . The references are very few. This manuscript has the potential to include more than 30 references.**

**Response to Reviewer #1**

Thank you for your constructive feedback. We have carefully addressed all your suggestions to improve the clarity and organization of our manuscript. Below are the specific changes we have made:

**1. Manuscript Structure Reorganization:**

- We have restructured the manuscript to include a clear **"Introduction"** section as the opening, which provides the background and motivation for our research.
- Following your suggestion, we have created a separate **"Related Work"** section immediately after the Introduction, which comprehensively reviews existing ECG classification and generation methods.
- The previous "Conclusions and Technical Limitations" has been renamed to **"Discussions"** and substantially expanded with more detailed analysis of our findings, comparisons with existing methods, and technical limitations.
- The **"Conclusion"** section has been condensed to two concise paragraphs, including future work directions.

**2. Enhanced Section Titles:** We have revised all section titles to be more precise and descriptive:

- "Introduction" - clearly establishes the research context
- "Related Work" - systematically reviews existing literature
- "Methodology and Technical Contributions" - details our approach
- "Data Preprocessing and Feature Analysis" - describes data handling
- "Network Architecture Design" - explains the PCA-CGAN structure
- "Experimental Results and Analysis" - presents findings
- "Discussions" - provides in-depth analysis and limitations
- "Conclusion" - summarizes key contributions and future directions

**3. Expanded References:** We have significantly expanded our reference list from the original count to **32 references**, incorporating more recent and relevant works in ECG classification, generative models, and data augmentation techniques.

**4. Enhanced Discussion Section:** The Discussions section now includes:

- In-depth analysis of main research achievements and contributions
- Detailed comparison with state-of-the-art methods
- Theoretical innovation and methodology paradigm shifts
- Technical breakthroughs in cross-patient generalization
- Multidimensional performance advantage analysis
- Comprehensive discussion of technical limitations and theoretical boundaries

We believe these revisions have significantly improved the manuscript's organization, clarity, and academic rigor. Thank you again for your valuable suggestions that helped strengthen our work.
